# Transparent and flexible passivation of MoS_2_/Ag nanowire with sputtered polytetrafluoroethylene film for high performance flexible heaters

**DOI:** 10.1038/s41598-022-09813-6

**Published:** 2022-04-09

**Authors:** Seung-Gyun Choi, Hae-Jun Seok, Jihyun Kim, Joohoon Kang, Han-Ki Kim

**Affiliations:** School of Advanced Materials Science and Engineering, Sungkyunkwan University, Suwon, Gyeonggi-do 16419 South Korea

**Keywords:** Materials science, Electronic properties and materials, Two-dimensional materials

## Abstract

We demonstrated highly transparent and flexible polytetrafluoroethylene (PTFE) passivation for the MoS_2_/Ag nanowire (Ag NW) electrodes used in thin film heaters (TFHs). The electrical, optical, and mechanical properties of PTFE coated MoS_2_/Ag NW electrode were compared to the bare MoS_2_/Ag NW electrode to demonstrate effective passivation of the sputtered PTFE films before and after the 85 °C–85% temperature-relative humidity environment test. In addition, we investigated the performances of TFHs with PTFE/MoS_2_/Ag NW as a function of PTFE thickness from 50 to 200 nm. The saturation temperature (87.3 °C) of TFHs with PTFE/MoS_2_/Ag NW electrode is higher than that (61.3 °C) of TFHs with bare MoS_2_/Ag NW, even after the 85 °C–85% temperature-relative humidity environment test, due to effective passivation of the PTFE layer. This indicates that transparent PTFE film prepared by sputtering process provides effective thin film passivation for the two-dimensional (2D) MoS_2_ and Ag NW hybrid electrode against harsh environment condition.

## Introduction

Thin film heaters (TFHs) used in smart windows for automobiles and smart buildings are basically operated by Joule heating of electrode when a direct current (DC) voltage is applied to the electrodes^1–6^. Recently, high performance TFHs have been used as heating sources for wearable devices, functional windows in smart building, and heaters for transparent windows in automobiles^2,7–11^. To improve the performance of TFHs, the development of high-quality transparent conductive electrodes (TCEs) with high conductivity, high optical transparency, and outstanding mechanical stability is imperative^12–15^. To date, Sn-doped In_2_O_3_ (ITO) prepared by physical vapor deposition or F-doped SnO_2_ (FTO) prepared by chemical vapor deposition are mainly used as TCE for rigid TFHs due to their high conductivity and optical transparency^16–20^. However, they have critical disadvantages such as high material cost and brittleness, due to specific ceramic characteristics of typical ITO and FTO films^2,21^. Therefore, to make flexible and transparent TFHs that could be applied in curved surface or wearable devices, it is necessary to develop flexible TCE as a substitute for the current ITO and FTO electrodes. As promising candidates to replace ITO and FTO films, conducting polymer, graphene, carbon nanotubes (CNT), metal mesh, polymer and oxide/metal/oxide (OMO) have been reported^3,13,22–25^. However, such TCEs to replace commercial ITO and FTO electrodes still have limitations. In case of the conducting polymer, it has critical problems such as low conductivity, chemical instability against oxygen ambient, and easy degradation under high temperature. Carbon-based conductive materials such as CNT and graphene showed a fairly high sheet resistance and fabricated by complicate process^26^. Both metal mesh and OMO electrode required high fabrication cost because they were fabricated by using vacuum-based sputtering process^27^. Another candidate to replace ITO and FTO electrode is the Ag nanowire (Ag NW) network prepared by printing process, because the Ag NW network offers low sheet resistance, high transmittance, outstanding flexibility, and a facile fabrication process^28–31^. However, Ag NW is vulnerable to the external environment, because Ag NW is degraded by oxidation with ambient O_2_ and H_2_O^32,33^. In addition, the Ag NW-based TFH has low operating stability against electrical and thermal stress during device operation^34,35^. To overcome this problem, an additional functional material should be coated on the Ag NW network, such as organic or inorganic layers^36–39^. Molybdenum disulfide (MoS_2_), which is a transition metal dichalcogenide, is basically a two-dimensional (2D) material, and has been applied to various applications due to its high surface-to-volume ratio, large optical absorption and relatively high thermal stability^40–45^. As a result, it is possible to enhance the thermal dispersion of the Ag NW network and decrease the thermal stress of the Ag NW junction by coating a 2D MoS_2_ layer on the Ag NW junction. However, the 2D MoS_2_ layer is a highly hygroscopic material and has high surface energy, whereby the absorption of oxygen and water is extremely high^46–49^. Therefore, the 2D MoS_2_ layer over coating on Ag NW network leads to absorption of H_2_O or O_2_ molecules, and despite the several advantages of the 2D MoS_2_ layer, results in degradation of the Ag NW network. As the market of TFHs applications tentatively increases, the high stability and reliability of TCE used in TFHs become more important. But, when the MoS_2_/Ag NW used in TFHs is exposed to the external and harsh environment, such as variable external temperature, oxygen, and H_2_O, the electrical and optical properties of MoS_2_/Ag NW and the performance of TFHs are gradually degraded^32,50–55^. To overcome this issue, the operating stability of the MoS_2_/Ag NW structure can be enhanced by using a polytetrafluoroethylene (PTFE) coating as a passivation layer. The sputtered PTFE film is currently being studied in many research areas such as flexible solar cells, anti-icing glasses, electromagnetic shield, and TFHs due to its superior hydrophobic, anti-reflection, or and passivation characteristics^56–59^. The PTFE layer with good temperature stability and high hydrophobic properties can protect the MoS_2_/Ag NW electrodes. However, to the best of our knowledge, there have been no reports on the thin film passivation of PTFE film for MoS_2_/Ag NW electrode to improve the stability of MoS_2_/Ag NW-based TFHs.

In this study, we report the characteristics of MoS_2_ coated Ag NW electrodes and thin film of superior passivation of sputtered PTFE to protect MoS_2_/Ag NW electrodes. The electrical, optical, and mechanical properties of the PTFE/MoS_2_/Ag NW electrodes and the bare MoS_2_/Ag NW electrodes were compared to confirm the effective passivation of the PTFE layer. To show the feasibility of the PTFE passivation layer, we compare the performance of flexible and transparent TFHs with PTFE/MoS_2_/Ag NW electrodes and bare MoS_2_/Ag NW electrode after 85 °C–85% temperature-relative humidity environment test. Based on the performance of the flexible and transparent TFHs, we suggest the potential of sputtered PTFE passivation layer to use on the MoS_2_/Ag NW hybrid electrodes in TFHs for smart windows.

## Results

**Figure 1:**
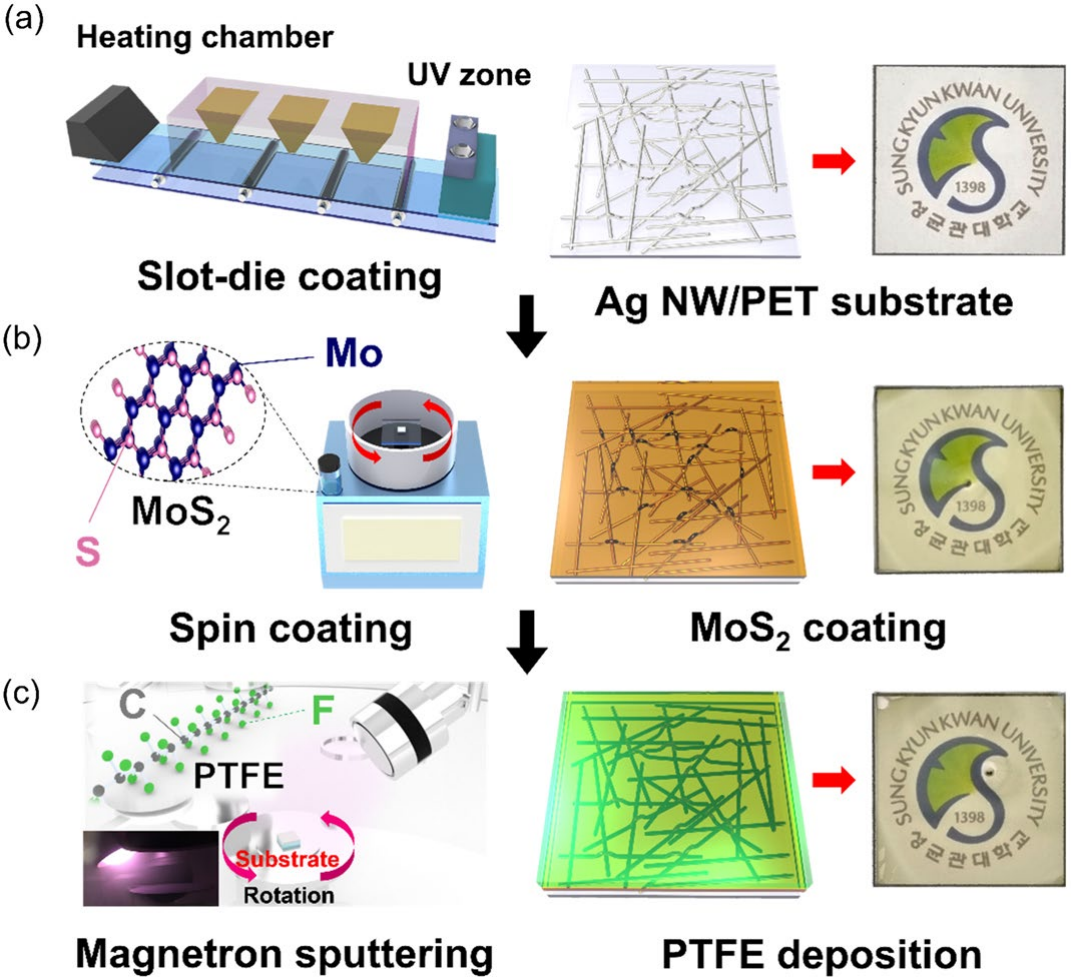
Schematic of the consecutive fabrication of PTFE/MoS_2_/Ag NW film in the following order (**a**) slot-die coating of Ag NW film, (**b**) spin coating of MoS_2_ crystals, and (**c**) RF magnetron sputtering using a 4-in. PTFE target.

Figure 1a,b show the fabrication processes of the slot-die coating of Ag NW film and spin-coated 2D MoS_2_ layer on the Ag NW film. Also, Fig. 1c shows a schematic of the RF magnetron sputtering of PTFE films on MoS_2_/Ag NW sample using PTFE target at room temperature. Specifically, we fabricated PTFE/MoS_2_/Ag NW samples depending on the thickness of PTFE to compare the optimized passivation effect with electrical and optical properties. The samples were indicated by different layers of the bare Ag NW (#1), MoS_2_/Ag NW (#2), and PTFE/MoS_2_/Ag NW as a function of PTFE thickness (#3: 50 nm, #4: 100 nm, #5: 150 nm, #6: 200 nm), respectively.

**Figure 2:**
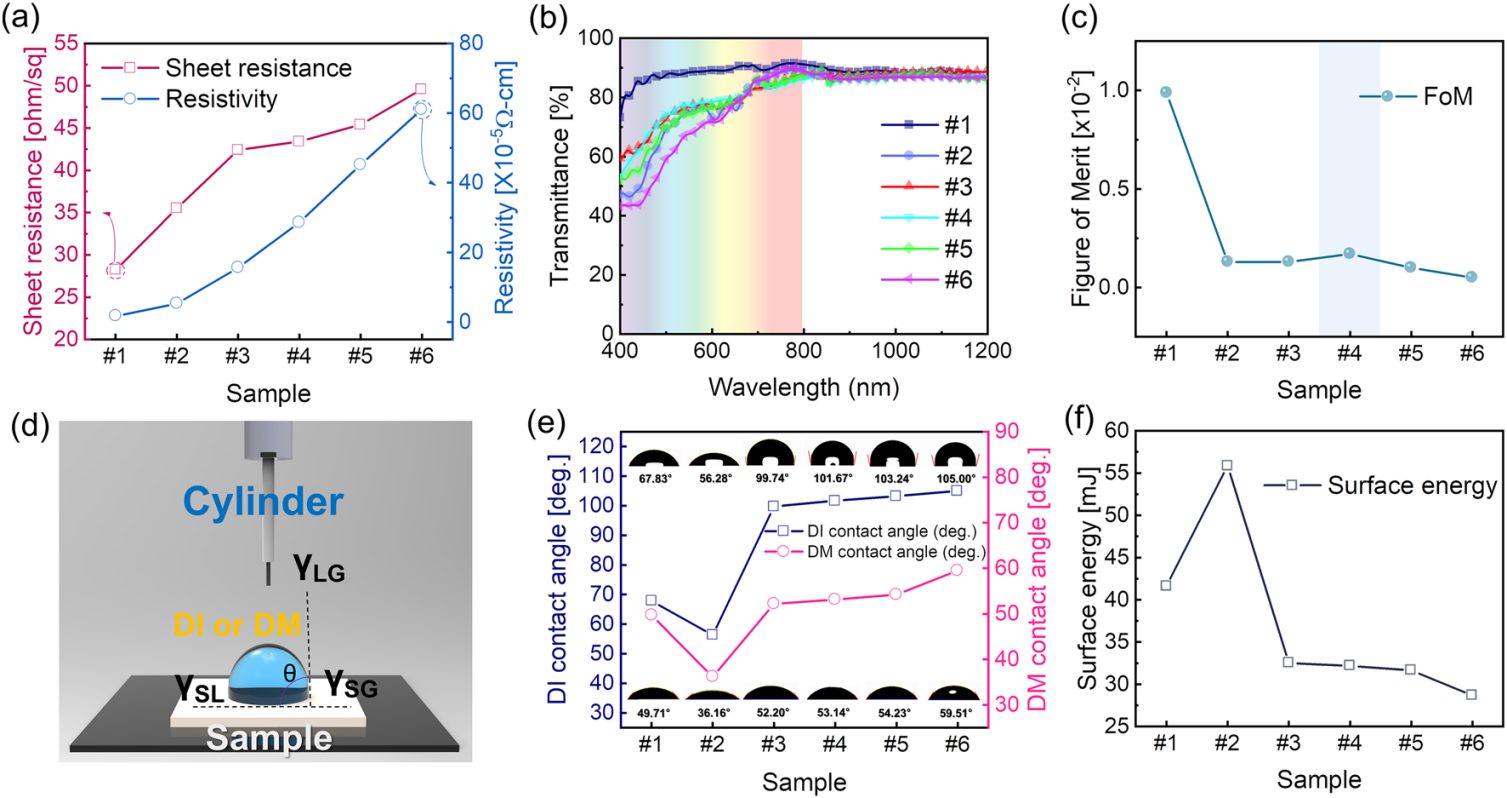
(**a**) Sheet resistance and resistivity from Hall measurement, (**b**) Optical transmittance of the different films in the wavelength (400–1200) nm. (**c**) The Figure of Merit values calculated from the sheet resistance and optical transmittance of the different films. (**d**) Schematic of the contact angle measurement system. (**e**) The calculated contact angle of the bare Ag NW, MoS_2_/Ag NW, and PTFE/MoS_2_/Ag NW samples using de-ionized water and diiodomethane. The Inset images show the captured shapes of droplet on the different samples depending on de-ionized water (top), and diiodomethane (bottom). (**f**) Calculated surface energy of the bare Ag NW, MoS_2_/Ag NW, and PTFE/MoS_2_/Ag NW samples as a function of the PTFE thickness.

Figure 2a shows the sheet resistance and resistivity of the samples that were measured using Hall measurement. With increasing sample number from #1 to #6, the sheet resistance increased from (28.2 to 49.6) Ohm/sq, and resistivity also increased from (1.69 to 61.1) × 10^–5^ Ω-cm. As the thickness of the PTFE film became thicker, the electrical resistance increased, because the MoS_2_ and PTFE layers had high resistivity. Figure 2b shows the optical transmittance of the various samples depending on the wavelength region from (400 to 1200) nm. The bare Ag NW sample showed a high transmittance of 88.23% at the visible wavelength region of (400–800) nm. When each MoS_2_ layer was coated on the Ag NW, there was no change in the optical transmittance due to the high optical transmittance of the 2D-MoS_2_. However, the sputtering of PTFE layer on the MoS_2_/Ag NW sample led to decrease of the optical average transmittance in the wavelength region < 600 nm with increasing PTFE layer thickness. Table 1 summarizes the details of the electrical and optical properties with the bare Ag NW, MoS_2_/Ag NW, and PTFE/MoS_2_/Ag NW film with various PTFE thickness. To determine the optimal thickness of the sputtered PTFE layer, the Figure of Merit (*FoM*) values were calculated from the sheet resistance (R_s_) and optical transmittance (T_av_) as shown in Fig. 2c, according to the following equation^60^:

**Table 1:** The optical and electrical properties of the bare Ag NW, MoS_2_/Ag NW and PTFE/MoS_2_/Ag NW film depending on the PTFE thickness.

Sample	Sheet resistance [ohm/sq]	Resistivity [× 10^−5^ Ω-cm]	Transmittance at 550 nm [%]	Average transmittance at visible region of (400–800 nm) [%]
#1	28.2	1.69	90.65	88.23
#2	35.5	5.27	80.69	73.69
#3	42.4	15.6	80.41	74.88
#4	43.4	28.6	81.08	76.60
#5	45.4	45.2	80.55	73.54
#6	49.6	61.1	80.16	70.09

1 $$Figure \; of \; Merit \; \left(FoM\right)=\frac{{T}_{av}^{10}}{{R}_{s}}$$

Although the PTFE/MoS_2_/Ag NW samples showed lower *FoM* values than the bare Ag NW or MoS2/Ag NW samples, all PTFE/MoS_2_/Ag NW sample showed similar *FoM* values, indicating that the PTFE thickness did not affect the electrical or optical properties of the electrodes. Among the PTFE/MoS_2_/Ag NW samples, the PTFE (100 nm)/MoS_2_/Ag NW showed the highest *FoM* value. To compare the surface properties of samples, we measured the contact angle and surface energy of the samples. Figure 2d shows a schematic of the contact angle measurement system using de-ionized water and diiodomethane liquid droplets. The contact angle was calculated from the angle of the interface between the film and the liquid when the liquid was dropped onto the surface of the sample. Figure 2e shows the contact angle depending on de-ionized water and diiodomethane droplets to calculate the surface energy of samples from #1 to #6. Table 2 summarizes the estimated contact angle and surface energy depending on the different liquid. The contact angle of liquid droplets on the thin film surface was determined as the following Young’s equation^61^:

**Table 2:** The measured contact angle and surface energy of the bare Ag NW, MoS_2_/Ag NW and PTFE/MoS_2_/Ag NW samples with different PTFE thickness.

Sample	De-ionized water [°]	Diiodomethane [°]	Surface energy [mJ]
#1	67.83	49.71	41.61
#2	56.28	36.16	55.89
#3	99.74	52.20	32.52
#4	101.67	53.14	32.18
#5	103.24	54.23	31.65
#6	105.00	59.51	28.66

2 $${\gamma }_{LG}\mathit{cos} \, \theta ={\gamma }_{SG}-{\gamma }_{SL}$$

where, *γ*_*LG*_ is the interface free energy between liquid and gas; *γ*_*SG*_ is the interface free energy between solid and gas; *γ*_*SL*_ is the interface free energy between solid and liquid, and *θ* is the contact angle. Each liquid/gas/solid interface free energy can determine the contact angle with the surface. The contact angle of the bare Ag NW in de-ionized water droplets was 67.83°. When the MoS_2_ layer was coated on the Ag NW, the contact angle decreased to 56.28° due to the high area ratio of 2D MoS_2_ with hydrophilic surface. This indicated that a coating of 2D MoS_2_ cannot protect the degradation of Ag NW network from the external environment. To overcome this problem, we directly sputtered a PTFE layer as a passivation layer. As a result, as the PTFE thickness increased from (50 to 200) nm, the contact angle tended to slightly increase, due to the hydrophobic surface of the PTFE film. In the case of the diiodomethane droplet, the contact angle of the bare Ag NW was 49.71°, and the angle of the MoS_2_ layer was further lowered to 36.16°. As mentioned above, when the PTFE layer was deposited, the contact angle gradually increased from (52.20 to 59.51)°. Therefore, the sputtered passivation PTFE layer changed the surface of MoS_2_/Ag NW from hydrophilic to hydrophobic, which is beneficial to prevent MoS_2_/Ag NW electrodes from the ambient condition. Figure 2f shows the calculated values of the surface energy from the contact angle from deionized water and diiodomethane depending on the samples. The surface free energy is calculated using the Owens–Wendt method, and can be calculated by the following equation^62^:

3 $${\gamma }_{s}={\gamma }_{s}^{d}+{\gamma }_{s}^{p}$$

4 $${{(\gamma }_{s}^{d})}^{0.5}=\frac{{\gamma }_{d}\left(cos{\theta }_{d}+1\right)-\sqrt{\left( {{{\gamma _d^p} / {\gamma _w^p}}} \right)}{\gamma }_{w}(cos{\theta }_{w}+1)}{2\left(\sqrt{{\gamma }_{d}^{d}}-\sqrt{{\gamma }_{d}^{p}\left( {{{\gamma _w^p} / {\gamma _w^p}}} \right)}\right)}$$

5 $${\left({\gamma }_{s}^{p}\right)}^{0.5}= \frac{{\gamma }_{w}\left(cos{\theta }_{w}+1\right)-2\sqrt{{\gamma }_{S}^{d}{\gamma }_{w}^{d}}}{2\sqrt{{\gamma }_{w}^{p}}}$$

In Eq. (3), ${\gamma }_{s}$ is the surface free energy, ${\gamma }_{s}^{d}$ is the dispersion component of surface free energy, and ${\gamma }_{s}^{p}$ is the polar component of surface free energy. Consequently, the ${\gamma }_{s}^{d}$ and ${\gamma }_{s}^{p}$ are estimated using the following Eqs. (4) and (5), respectively, where ${\gamma }_{d}$ is the surface free energy of diiodomethane, ${\gamma }_{d}^{d}$ is the dispersive component of diiodomethane surface energy, ${\gamma }_{d}^{p}$ is the polar component of water surface energy, ${\gamma }_{w}$ is the surface free energy of the de-ionized water, ${\gamma }_{w}^{d}$ is the dispersive component of de-ionized water surface free energy, and ${\theta }_{d}$ and ${\theta }_{w}$ are the contact angles of diiodomethane and de-ionized water, respectively. As a result, the MoS_2_/Ag NW sample showed the highest surface energy of 55.89 mJ. As the thickness of the PTFE layer increased, the surface energy of samples slightly decreased. Therefore, this verified that the PTFE layer could act as a the passivation layer that could sufficiently withstand the external environment^63,64^.

**Figure 3:**
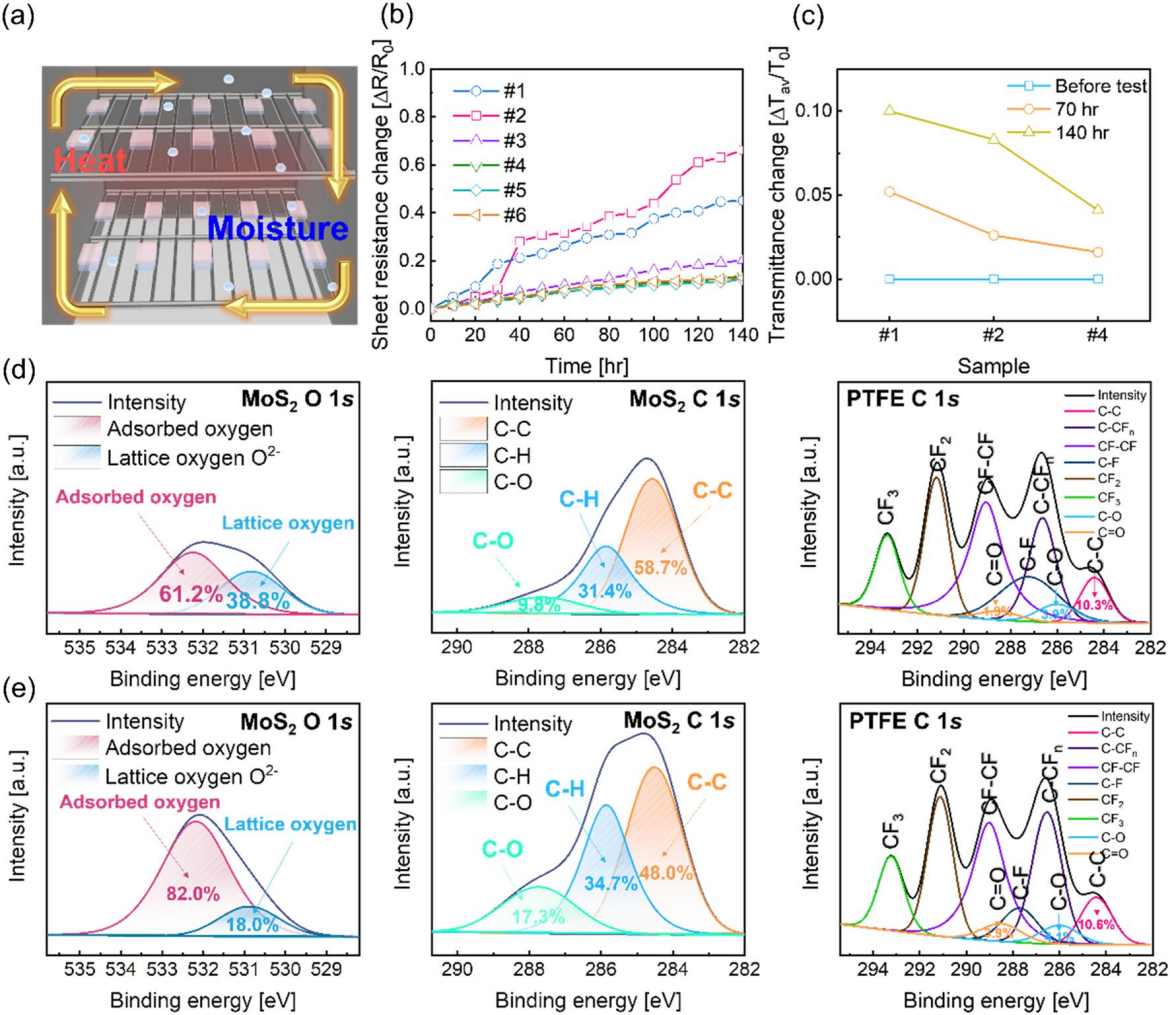
(**a**) Schematic of the 85 °C–85% temperature-humidity environment test system. (**b**) The change of sheet resistance and (**c**) optical average transmittance in the visible region from (400 to 800) nm obtained from the bare Ag NW, MoS_2_/Ag NW, and PTFE/MoS_2_/Ag NW samples during the temperature-humidity environment test. XPS analysis showing the O 1*s* core peak deconvoluted into two adsorbed oxygen and lattice oxygen, and the C 1*s* peak deconvoluted into three C–C, C–H, and C–O peaks in the 2D MoS_2_ film and C 1*s* spectra of the sputtered PTFE layer on the MoS_2_/Ag NW film (**d**) before, and (**e**) after 85 °C–85% temperature-relative humidity environment test.

To investigate the passivation effect of the sputtered PTFE film, we conducted an external environment test with each sample and Fig. 3a shows a schematic of the 85 °C–85% temperature-relative humidity environment test system. Figure 3b shows that the change of sheet resistance of the bare Ag NW, MoS_2_/Ag NW, and PTFE/MoS_2_/Ag NW samples in the 85 °C–85% temperature-relative humidity environment. The changes in the electrical sheet resistance characteristics of each sample were measured by four-point probe device during the environment test. The test was performed every 10 h and then the sheet resistance was measured and repeated for 140 h. In the case of the bare Ag NW, its sheet resistance increased due to the oxidation of Ag NW, the adsorption of H_2_O, and the sulfurization of Ag NW. Also, the sheet resistance of the MoS_2_/Ag NW sample showed significantly higher than that of the bare Ag NW sample. This clearly showed that the MoS_2_ layer was not suitable for the passivation layer of Ag NW. The resistance changes of the PTFE/MoS_2_/Ag NW samples were also evaluated as a function of the test time. Compared to the bare Ag NW and MoS_2_/Ag NW samples, the PTFE coated sample showed little change in sheet resistance. Due to the hydrophobic and high thermal stability of the PTFE passivation layer, there was no significant change in resistance, even after the 85 °C–85% temperature-relative humidity environment test for 140 h. Figure 3c shows the change of optical average transmittance in the visible wavelength region between (400 and 800) nm with various samples during the 85 °C–85% temperature-relative humidity environment test. The passivation test was conducted for total of 140 h, and the optical transmittance of all samples was measured every 70 h. Figure S1 shows the optical transmittance of each sample at the (400–800) nm region depending on the environment test. The average optical transmittance of the bare Ag NW sample was 88.23% in the visible wavelength region before the passivation test of 140 h. Thereafter, the average optical transmittance of the bare Ag NW after the 140 h passivation test was slightly decreased to 79.38%. Moreover, when MoS_2_ was coated on the Ag NW sample, the optical average transmittance decreased from (73.69 to 67.60) % in the visible wavelength region during harsh environment test. This confirmed that the optical transmittance severely decreased due to the influence of the hygroscopic property of the 2D MoS_2_ layer. In the case of the PTFE/MoS_2_/Ag NW samples, the transmittance did not significantly decrease after the passivation test regardless of the thickness of the PTFE due to the hydrophobic properties and thermal stability of the PTFE. To investigate the chemical composition of the MoS_2_ layer on the Ag NW and PTFE layer on MoS_2_/Ag NW, the core level spectra change was examined through XPS analysis before and after the 85 °C–85% temperature-relative humidity environment test of 140 h. We calibrated all binding energies of peaks through the C (carbon) 1*s* peak at 284.8 eV. Figure 3d,e show the XPS results of the O 1*s*, and C 1*s* peaks of MoS_2_ layer on Ag NW film and the C 1*s* peak of the PTFE layer on the MoS_2_/Ag NW film before and after the temperature-relative humidity environment test, fitting through the Gaussian function. The position of the O 1*s* core peak is 532.0 eV, and it was deconvoluted into two other peaks of chemical binding energy in the lattice oxygen (Mo–O bonding) (O_I_) peak at 530.8 eV, and adsorbed oxygen (chemical absorbed oxygen and –OH group) (O_II_) peak at 532.3 eV^65^. Due to the hygroscopic properties of MoS_2_, the adsorbed oxygen peak area ratio relatively increased, compared to that of the lattice oxygen. To calculate the change in ratio of O_I_ and O_II_, the peak area ratios were evaluated for O_II_/(O_I_ + O_II_); the peak area ratio of O 1*s* at MoS_2_ increased from (61.2 to 82.0) %. The C 1*s* spectra were deconvoluted into three peaks of carbon–carbon (C–C) bonding of C_I_ peak at 284.6 eV, carbon-hydrogen (C–H) bonding of C_II_ peak at 285.9 eV, and carbon–oxygen (C–O) bonding of C_III_ peak at 287.6 eV. Because of the high moisture and temperature during harsh environment test, the C_II_ and C_III_ peak area ratio relatively increased, compared to the C_I_ peak. As a result, the MoS_2_ layer was not suitable as a passivation layer for the specific severe environment. Eventually, the electrical resistance of the MoS_2_/Ag NW sample increased during the temperature-humidity environment test. The C 1*s* spectrum of the PTFE layer was deconvoluted into different peaks consisted of distinct groups: The C–C bonding of peak at 284.6 eV; the C-CF_n_ bonding of peak at 286.6 eV; the C–F bonding peak at 287.2 eV; the CF–CF bonding peak at 289.1 eV; the CF_2_ bonding peak at 291.2 eV; the CF_3_ bonding peak at 293.3 eV^49^. In particular, there was a carbon–oxygen (C–O) single bonding peak at 285.9 eV, and a carbon=oxygen (C=O) double bonding peak at 288.6 eV. After the external environment test, the area ratio of C–C, C–O, and C=O peaks with PTFE C 1*s* increased little, compared to before the test. This indicated that due to the passivation effect of PTFE, the oxidation and adsorbing functional groups at the PTFE surface were hardly observed in the C-F groups due to the interactions of the C-F bonding^66–68^. Consequently, the sputtered PTFE film can act as an effective passivation layer for stable TFH, even in a harsh external environment.

**Figure 4:**
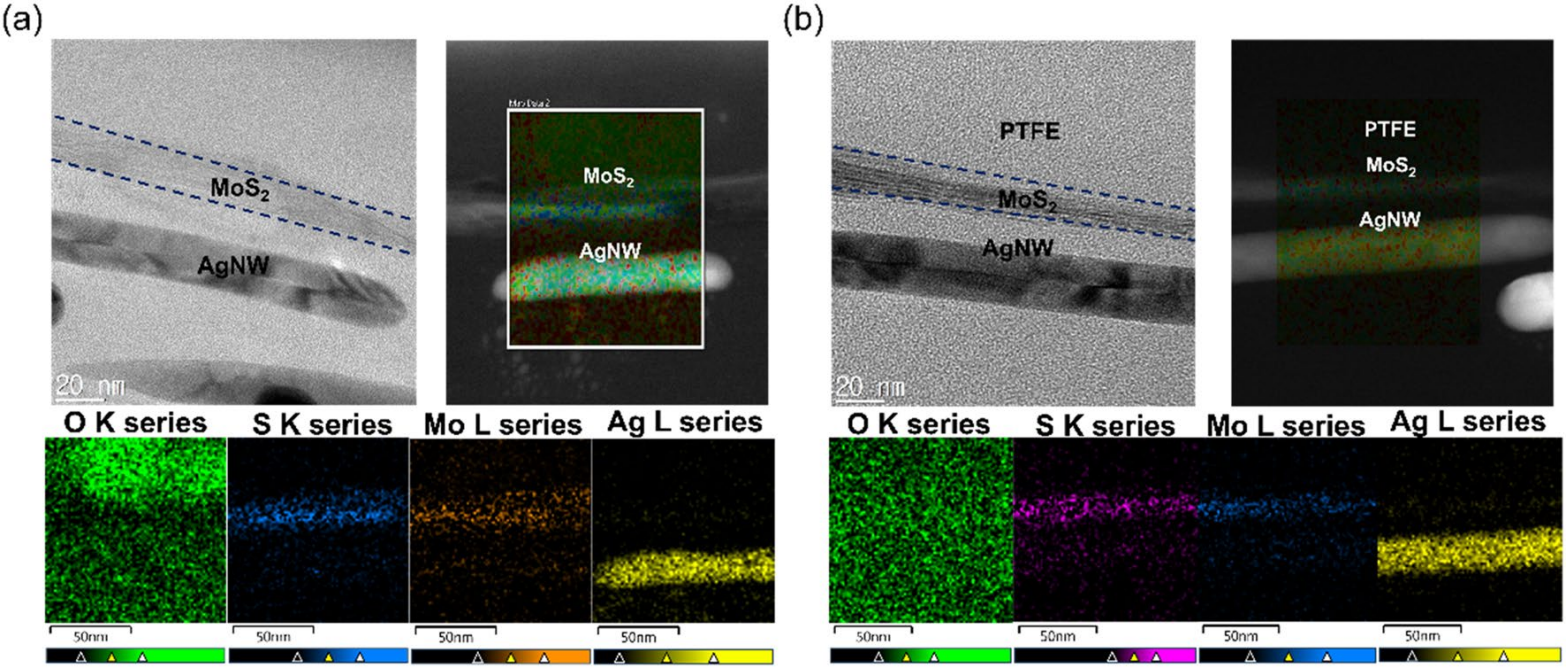
Enlarged cross-sectional images and EDS mapping images with O, S, Mo, and Ag elements obtained from HR-TEM for (**a**) the MoS_2_/Ag NW and (**b**) PTFE (100 nm)/MoS_2_/Ag NW film after environmental test under 85 °C–85% temperature-relative humidity for 140 h.

HR-TEM was employed to investigate the microstructure of the Ag NW, MoS_2_, and PTFE layer after an 85 °C–85% external environment test to verify the passivation effect of the PTFE layer. Figure 4a shows a cross-sectional image and EDS mapping images of the bare MoS_2_/Ag NW electrode after the 85 °C–85% external environment test. This clearly shows that the oxygen adsorption increased under a harsh environment due to the hygroscopic properties of the 2D MoS_2_ layer. Also, the Mo and S elements were slightly dispersed because of the exposure to high-temperature conditions. Figure 4b shows the cross-sectional image and EDS mapping images of the PTFE/MoS_2_/Ag NW electrode. Unlike the bare MoS_2_/Ag NW electrode, the adsorption of the MoS_2_ layer was reduced by the passivation of the PTFE layer, and the dispersion of the Mo and S elements was also slightly decreased by the thermal stability of the PTFE layer. Consequently, the sputtered PTFE layer effectively protect the MoS_2_/Ag NW electrodes against the external environment.

**Figure 5:**
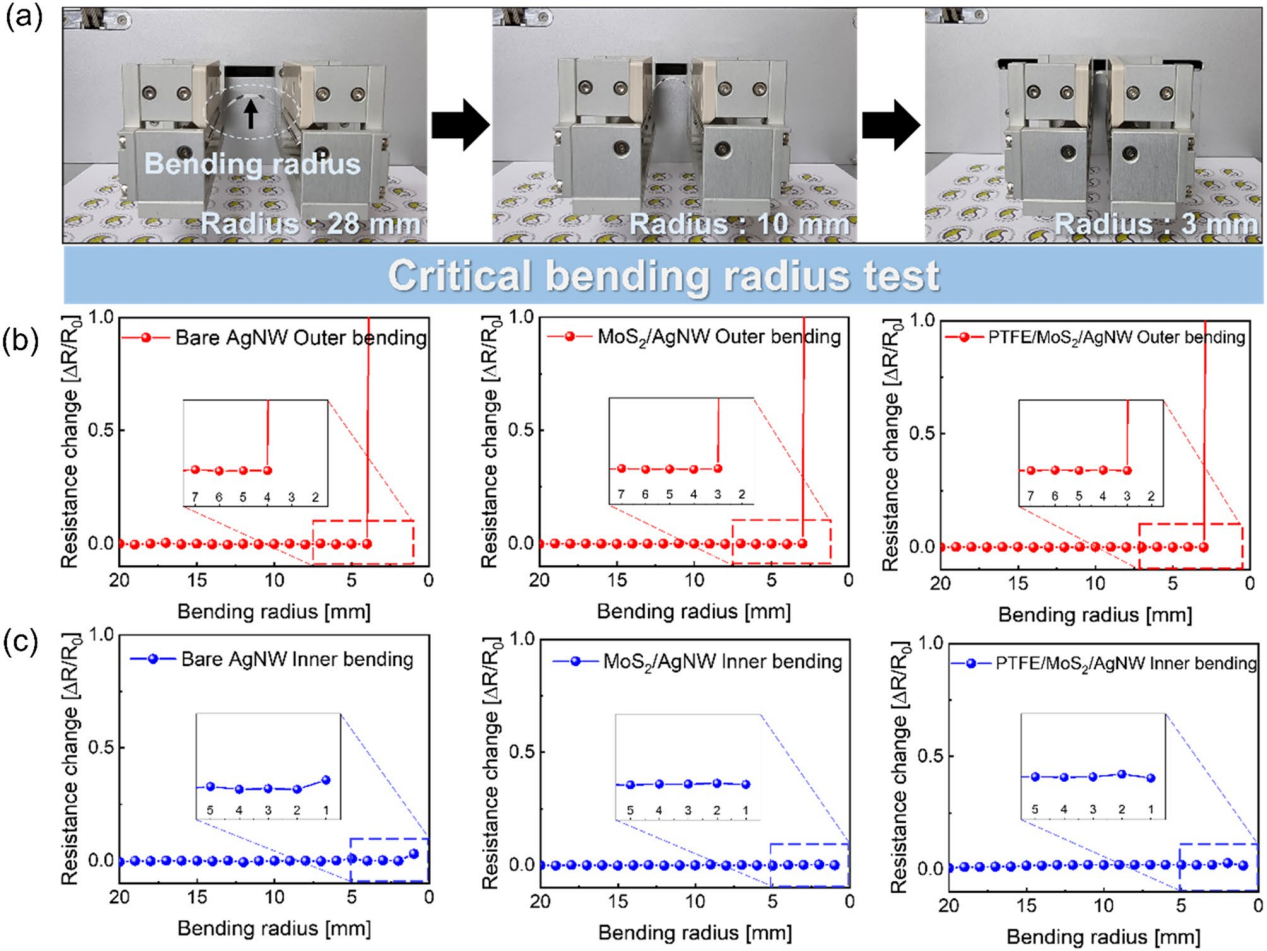
(**a**) Photographs of the operating bending test system depending on the bending radius to estimate the critical radius. (**b**) Outer critical bending radius results, and (**c**) inner critical bending radius results, for the bare Ag NW, MoS_2_/Ag NW, and PTFE (100 nm)/MoS_2_/Ag NW samples.

Figure 5a shows the bending test steps for the bare Ag NW, MoS_2_/Ag NW, and PTFE/MoS_2_/Ag NW on PET substrate having a size of 1.5 cm × 6.0 cm as a function of the bending radius using a bending test system. In outer bending test, the bending radius decreased with increasing mechanical stress on the thin film. Figure 5b shows the resistance change according to the outer bending test of the bare Ag NW, MoS_2_/Ag NW, and PTFE/MoS_2_/Ag NW, respectively. When tensile stress is applied to the thin film during outer bending, a particularly large change in resistance may occur. The change of resistance ($\Delta R)$ is defined as the following equation where the initial resistance is ${R}_{0}$, and the resistance $\left(R\right)$ is changed depending on the bending radius.

6 $$\Delta R=\left(R-{R}_{0}\right)/{R}_{0}$$

In addition, the critical radius is defined as the point at which as the bending radius decreases, the resistance change rapidly increases. The critical radius of the bare Ag NW samples at outer bending was 3 mm. In the case of the bare Ag NW, it was easily isolated, due to the tensile stress applied to the Ag NW. However, the critical radius of MoS_2_/Ag NW was 2 mm, showing a slightly lower radius than the bare Ag NW. The 2D MoS_2_ layer coated Ag NW can evenly cover Ag nanowires and junctions and can play a role in mitigating the mechanical stress applied to the film. This was related to the durability of the wire-wire junctions that determine the conductivity of the Ag NW. In addition, the PTFE/MoS_2_/Ag NW sample also showed a critical radius of 2 mm, indicating that the sputtered PTFE layer does not affect the mechanical flexibility of the MoS_2_/Ag NW electrodes. Figure 5c shows the resistance change along with the inner bending test of the bare Ag NW, MoS_2_/Ag NW, and PTFE/MoS_2_/Ag NW. In the case of inner bending, compressive stress was applied on the film, but the variation of the electrical properties of the film was smaller than that of the tensile stress. As a result, all samples showed a critical radius of 1 mm and demonstrated small electrical change, compared to outer bending.

**Figure 6:**
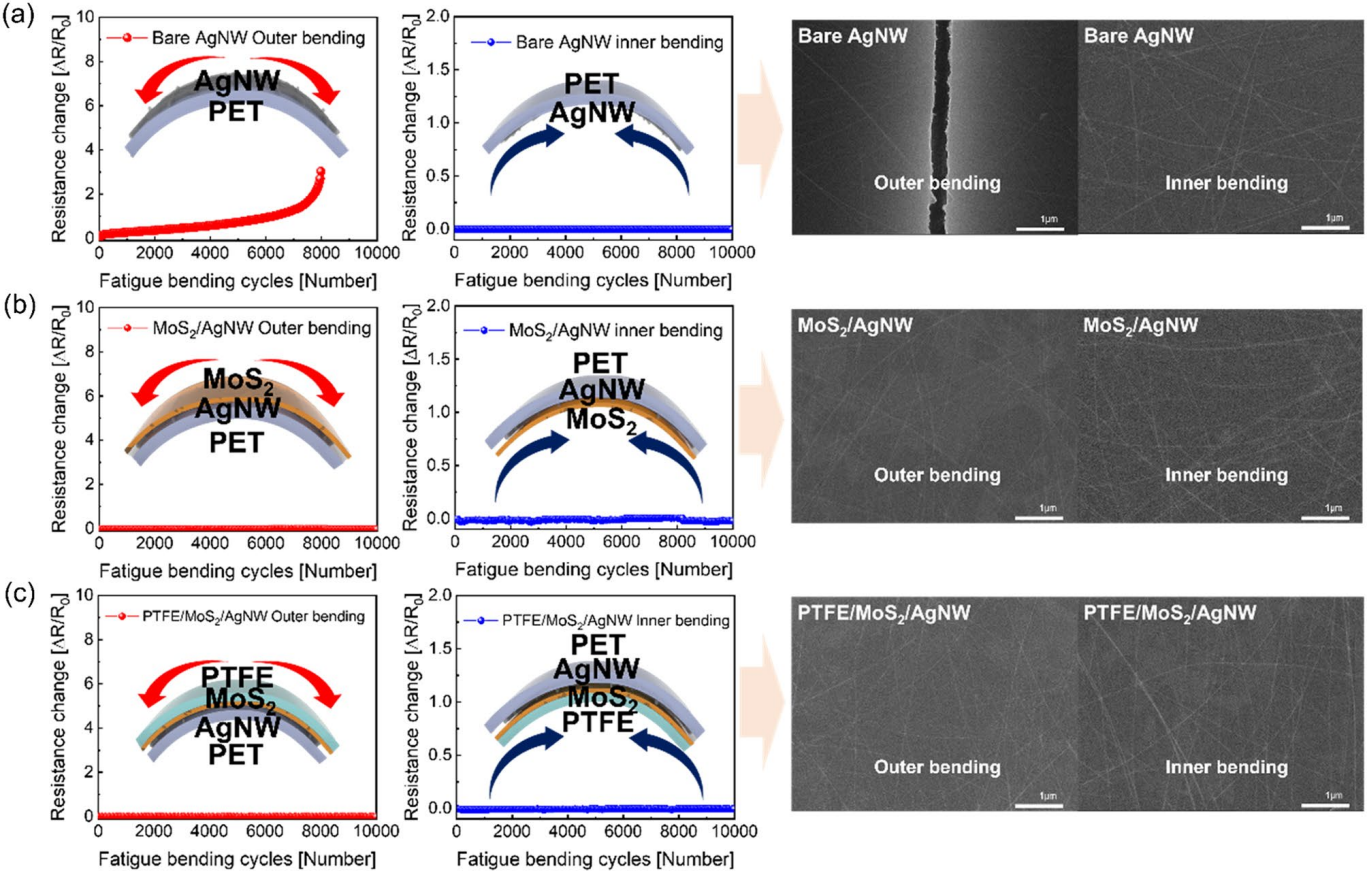
Dynamic fatigue test for the outer and inner bending cycle of (**a**) the bare Ag NW, (**b**) MoS_2_/Ag NW, and (**c**) PTFE (100 nm)/MoS_2_/Ag NW samples, for 10,000 cycles. The right side of the figure shows surface SEM images of the samples after the outer (left) and inner (right) fatigue test.

Figure 6 shows the change of electrical resistance through mechanical fatigue test of the bare Ag NW, MoS_2_/Ag NW, and PTFE/MoS_2_/Ag NW samples according to outer and inner bending with the fixed bending radius of 15 mm for 10,000 cycles. In the case of the bare Ag NW sample in Fig. 6a, the electrical resistance of the bare Ag NW sample tended to increase with increasing outer bending cycles. Mechanical stress repeatedly applied to the Ag NW during the outer bending fatigue test led to degradation of the Ag NW network. However, when the MoS_2_ and PTFE layers were coated as shown in Fig. 6b,c, the resistance did not change regardless of the bending mode. This proved that the additional coating layer could improve the durability and flexibility of the Ag NW electrode. The mechanical bending test results, it confirmed that the electrical stability of the Ag NW electrode can be improved through over coating of MoS_2_ and PTFE. The right side of Fig. 6 shows the surface FE-SEM image of the bare Ag NW, MoS_2_/Ag NW, and PTFE/MoS_2_/Ag NW samples after outer (left) and inner (right) fatigue cyclic test. After fatigue test, the SEM image of the outer fatigue bending cycles for the bare Ag NW sample showed the dissociation of film with separation of the Ag NW, because there was no over-coating film that could mitigate the mechanical stress of the Ag NW. However, the over-coating of the 2D MoS_2_ layer led to identical surface SEM image, even after 10,000 cycles fatigue test. Furthermore, it showed that the Ag NW junction was well maintained, without any cracks or nanowire disconnection. Surface SEM image of the PTFE/MoS_2_/Ag NW film also shows well-connected Ag NW network after 10,000 cycles fatigue test like the MoS_2_/Ag NW sample. As a result, this confirmed that the MoS_2_ over coating and sputtered PTFE layer on the Ag NW electrode improved the mechanical flexibility and stability of the Ag NW network by the bridge effect of the 2D MoS_2_ and over-coating of the flexible PTFE layer. Therefore, the cover coating of 2D MoS_2_ is beneficial for flexibility, and the sputtered PTFE layer is beneficial for the passivation of MoS_2_/Ag NW electrode.

**Figure 7:**
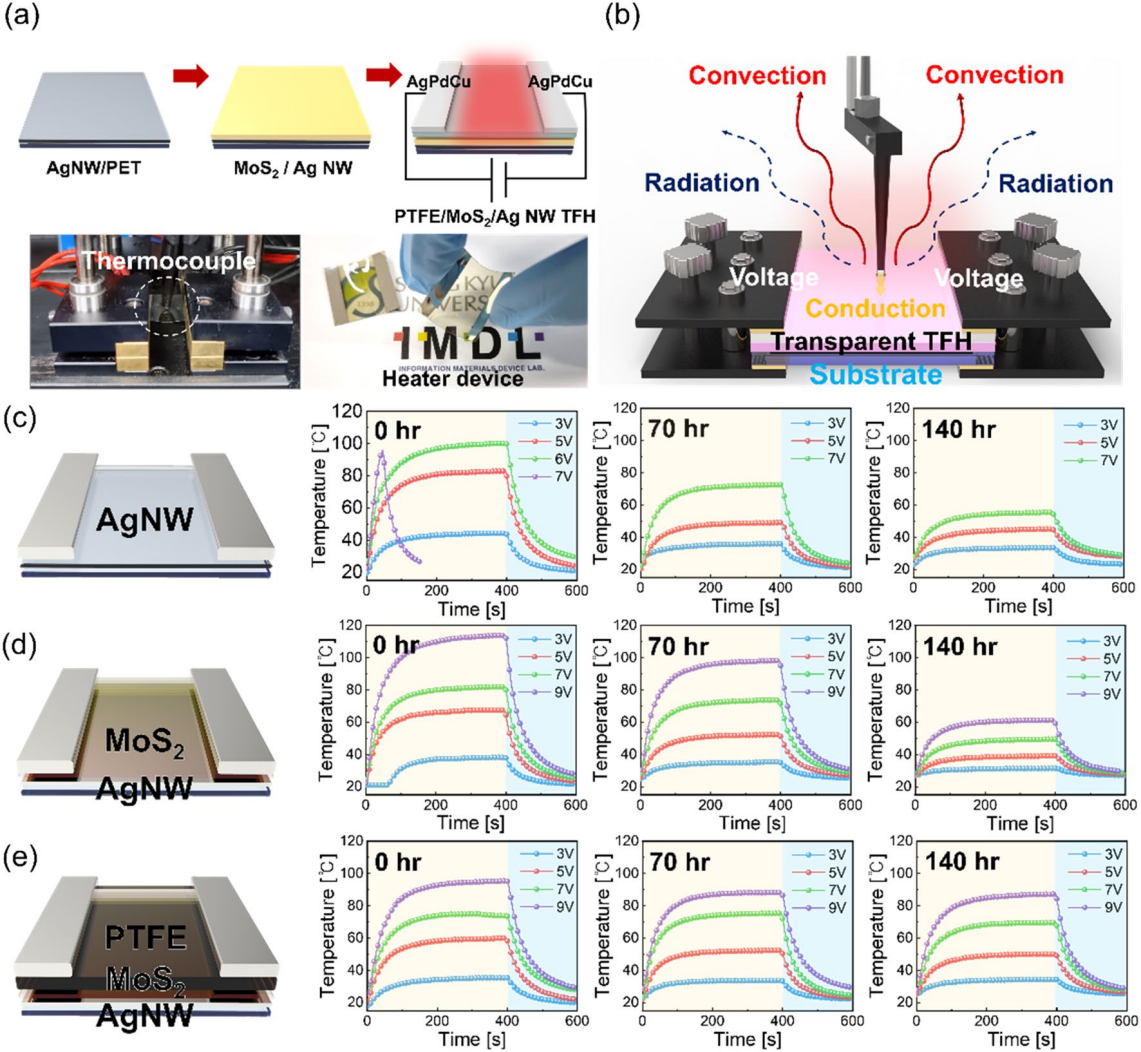
(**a**) Schematic of the fabrication process of the PTFE/MoS_2_/Ag NW TFHs; at bottom of shows the thermocouple that is used to analyse the saturation temperature of TFHs as a function of DC voltage. (**b**) Schematic of the Joule heating mechanism of TFH when the DC voltage was applied to TFHs. (**c**) Temperature profiles of the TFHs fabricated on the bare Ag NW, and (**d**) MoS_2_/Ag NW. (**e**) PTFE (100 nm)/MoS_2_/Ag NW electrode during the 85 °C–85% temperature-relative humidity environment test.

To demonstrate the feasibility of the superior passivation effect of the sputtered PTFE layer, we fabricated PTFE/MoS_2_/Ag NW based-TFHs with hydrophobic passivation PTFE layer and compared their performance with that of the MoS_2_/Ag NW based- and bare Ag NW-based TFHs under harsh environment. Figure 7a shows a schematic of the TFHs fabrication process with PTFE/MoS_2_/Ag NW electrodes. We examined the TFHs performance using the temperature measurement system with thermocouple mounted on the conductive film as a function of the input DC voltage. Figure 7b shows the possible Joule heating mechanism of the transparent TFHs. Current (*I*) flows through a conductive thin film (2D MoS_2_/Ag NW) that generates Joule heat, and its magnitude can be expressed as proportional to the product of ${I}^{2}$, electrical resistance $R$ and time $t$^69^. Also, heat dissipation that is generated around the conductive thin film could be explained by conduction, convection in air, and radiation mechanisms^70^. If the heat loss of the conduction effect to the substrate was neglected, the heat convection effect would become the main effect of heat dissipation, and this effect has the following equation^71^:

7 $${I}^{2}R=\left({m}_{1}{C}_{1}+{m}_{2}{C}_{2}\right)\frac{dT\left(t\right)}{dt}+ A\left({h}_{1}+{h}_{2}\right)\left(T\left(t\right)-{T}_{0}\right)+\sigma A\left({\varepsilon }_{1}+{\varepsilon }_{2}\right)\left({T\left(t\right)}^{4}-{T}_{0}^{4}\right)$$

The conductive film and substrate are indicated by subscripts 1 and 2, respectively, where $m$ is the mass of the material, $c$ is the specific heat capacity, $h$ is the convective heat transfer co-efficient, $A$ is the heating area; $\sigma$ is the Stefan-Boltzmann constant, $\varepsilon$ is the emissivity of the conductive film, $T\left(t\right)$ is the estimated temperature as a function of time, and ${T}_{0}$ is the initial temperature under the ambient condition. It was important to decrease heat dissipation by reducing the heat convection effect, and to increase the saturation temperature when the TFH was operated at low voltage. To calculate the saturation temperature of the conductive thin film as a function of DC voltage, it can be defined in the following equation^72^:

8 $${T}_{s}=\frac{{v}^{2}\Delta t}{R{h}_{conv}{A}_{conv}}+{T}_{0}$$

where ${h}_{conv}$ is the convective heat transfer coefficient, and ${A}_{conv}$ is the surface area where convection occurs. Also, ${T}_{s}$ is the saturation temperature, and ${T}_{0}$ is the initial temperature. As a result, the sheet resistance of the film was lower, while the saturation temperature value was higher. Figure 7c–e show the temperature profiles of the transparent TFHs with the bare Ag NW, MoS_2_/Ag NW, and PTFE (100 nm)/MoS_2_/Ag NW under 85 °C–85% temperature-relative humidity environment test as a function of the input DC voltage. Figure S2 shows the TFHs performance as a function of the thickness of the PTFE layer of (50, 150, and 200) nm. Also, Fig. S3 shows the performance of TFHs that can reach the maximum saturation temperature depending on the applied DC voltage and the calculated operating power value when the same 6 V was applied. The power $P$ was expressed depending on the resistance value of the conductive film based on the equation $P= {V}^{2}/R$. Table 3 summarizes the saturation temperature of TFHs with different electrodes at specific DC voltages when the environmental test was conducted for 140 h. The left side of Fig. 7c shows that the bare Ag NW-based TFHs have a saturation temperature of 94.7 °C at DC voltage of 6 V. However, when the DC voltage is above 7 V, deterioration of the TFH occurred at over 100 °C. Figure 7d shows that TFH performance with the MoS_2_/Ag NW electrode as a function of the input DC voltage. Due to the existence of the high resistance 2D MoS_2_ over layer on the Ag NW, the MoS_2_/Ag NW led to higher sheet resistance of the electrode than that of the bare Ag NW electrode. Despite the higher sheet resistance, the MoS_2_/Ag NW-based TFH showed a higher saturation temperature of 114.1 °C, even at the higher DC voltage of 9 V. Because the MoS_2_ layer can adequately disperse the thermal stress of Ag wire-wire junction, the MoS_2_/Ag NW based TFH can reach a higher saturation temperature than can the bare Ag NW TFHs. Figure 7e shows the performance of the TFHs fabricated on the PTFE/MoS_2_/Ag NW electrode as a function of the input DC voltage. This shows a saturation temperature of 95.5 °C at 9 V. Similarly, due to the insulating PTFE passivation layer, the PTFE/MoS_2_/Ag NW-based TFH shows a lower saturation temperature than the MoS_2_/Ag NW sample at the same applied DC voltage. The right-side panels of Fig. 7c–e show the temperature profiles of the TFHs after the 85 °C–85% test for (70 and 140) h. The right side of Fig. 7c shows the performance of the bare Ag NW-based TFH after the 85 °C–85% environmental test. Consequently, it shows the saturation temperature of 55.4 °C under 7 V after the environment test for 140 h. This degradation of Ag NW-based TFHs could be explained by the oxidation and sulfurization of the Ag NW network when exposed to an externally humid environment at high temperature. In addition, wire-wire junctions were vulnerable to harsh environment, which decreases the operating stability of TFH. The right side of Fig. 7d shows the temperature profiles of MoS_2_/Ag NW-based TFH after the 85 °C–85% environmental test. After exposure to harsh environment, the TFH showed the saturation temperature of 61.3 °C at the same 9 V due to its increased sheet resistance. In particular, the hygroscopic and oxidation properties of the 2D MoS_2_ layer under harsh environments mainly affected the deteriorative characteristics of the TFHs. However, the right side of Fig. 7e shows that the PTFE/MoS_2_/Ag NW-based TFH reached a saturation temperature of 87.3 °C under 9 V even after the harsh environmental test. In addition, we explained the improved lifetime of the PTFE/MoS_2_/AgNW TFHs using the linear Arrhenius curve like below^4^.

**Table 3:** The saturation temperature of the bare Ag NW-based, MoS_2_/Ag NW-based and PTFE/MoS_2_/Ag NW-based TFHs after the 85 °C–85% environmental test for 140 h.

Sample	Saturation temperature (℃)											
	0 h				70 h				140 h			
	3 V	5 V	7 V	9 V	3 V	5 V	7 V	9 V	3 V	5 V	7 V	9 V
#1	44.4	83.1	–	–	35.9	48.9	72.3	–	30.4	45.1	55.4	–
#2	38.2	67.4	81.9	114.1	35.3	52.3	73.6	98.3	31.4	39.1	49.5	61.3
#4	35.3	59.9	74.0	95.5	33.8	52.3	75.4	88.2	34.3	50.1	69.7	87.3

9 $$\mathit{ln}\left(\frac{1}{{t}_{f}}\right)=\mathit{ln}\left(A\right)-\frac{{E}_{a}}{kT}$$

10 $$AF=\frac{{t}_{{f}_{2}}}{{t}_{f1}}$$

where t_f_ is the operating failure time of TFH, A is the pre-exponential factor, E_a_ is the activation energy, *k* is the Boltzmann’s constant, T is the absolute temperature and AF is acceleration factor at various temperature. Using above equations, we compared the failure time of the PTFE/MoS_2_/Ag NW TFHs and bare Ag NW TFHs as shown in Fig. S4. Compared to the bare Ag NW-based TFHs, the PTFE/MoS_2_/Ag NW TFHs showed improved failure time even after harsh environment tests due to passivation effect of PTFE film. This indicates that the sputtered PTFE layer effectively suppressed the oxidation or sulfurization of the hygroscopic MoS_2_/Ag NW electrode under high temperature and humidity. Due to the effective passivation of the PTFE layer, the conductivity of the MoS_2_/Ag NW electrode can be successfully maintained, even in a harsh environment. As a result, the passivation PTFE layer led to consistent saturation temperature of TFH performance that was similar to that of the as-fabricated sample.

**Figure 8:**
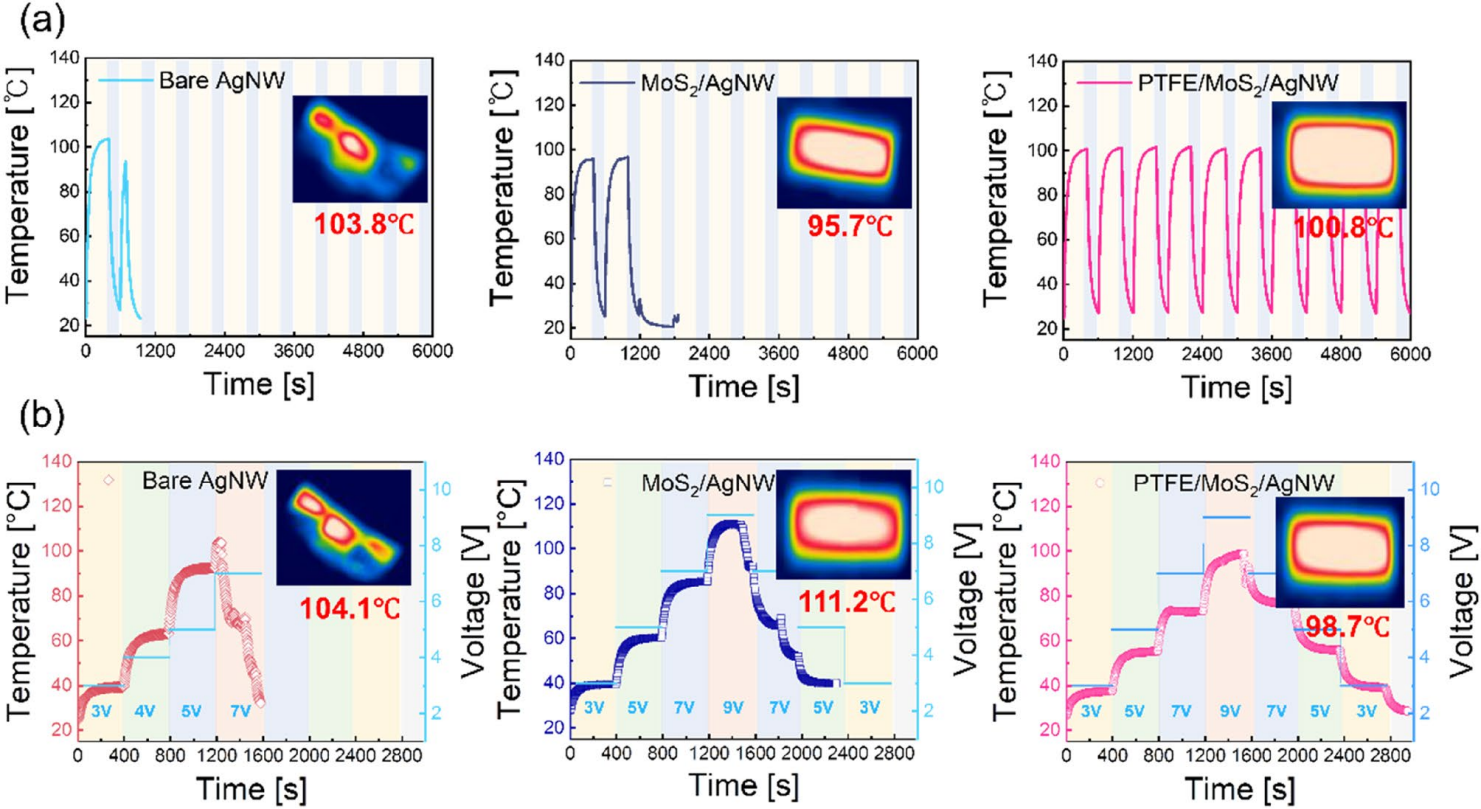
(**a**) Repeated cyclic test of the TFHs during 6000 s and (**b**) step test of the TFHs during 3000 s depending on the applied DC voltage of the bare Ag NW, MoS_2_/Ag NW, and PTFE/MoS_2_/Ag NW electrode. The inset IR images show the saturation temperature reached of the TFHs.

To investigate the operating stability of the Ag NW-based TFHs, we conducted operation stability test of the TFHs. Also, the inset images in each panel of Fig. 8 show IR images when the TFH reached saturation temperature, measured using the IR camera. Figure 8a shows the repeated temperature profiles of the bare Ag NW-based, MoS_2_/Ag NW-based, and PTFE/MoS_2_/Ag NW-based TFHs test in the wide temperature range to evaluate the repetitive on/off characteristics during 10 cycles. This clearly shows that the PTFE/MoS_2_/Ag NW-based TFHs exhibited superior operating stability under high saturation temperature at repeated on/off state compared with that of the bare Ag NW-based and MoS_2_/Ag NW-based TFHs. Figure 8b also shows the step test of the TFHs samples using consecutively applied different DC voltage without cooling step. Both the Ag NW-based, and MoS_2_/Ag NW TFHs show unstable cooling characteristics after the saturation temperature. Because the heat dispersion of the bare Ag NW-based and 2D MoS_2_/Ag NW-based electrode is not proper, there is no constant temperature step unlike the PTFE/MoS_2_/Ag NW-based TFH. The PTFE/MoS_2_/Ag NW TFH demonstrates, stable operational stability even after the saturation temperature because the thermal stress of the Ag NW junction is relieved by the MoS_2_ and PTFE layer. The outstanding performance and stability of PTFE/MoS_2_/Ag NW TFH indicates that the sputtered PTFE layer provides effective thin film passivation to fabricate high performance transparent and flexible TFHs for the next generation smart windows.

## Conclusions

We investigated the feasibility of sputtered PTFE film as passivation layer for 2D MoS_2_/Ag NW electrode to protect from harsh external environment, and provide operational stability of the TFHs due to the high hydrophobic and thermal properties of the PTFE layer. The performance of the bare Ag NW-based TFH was degraded at over DC voltage, because the Ag NW junctions were deteriorated by thermal stress. In addition, oxidation and sulfurization of the Ag NW network at high operational temperature led to degradation of the TFHs performance. Although the coating of 2D MoS_2_ nanosheet improved the thermal stability of the Ag NW electrode due to dispersal of Joule heat at the wire junctions, the hygroscopic 2D MoS_2_ led to the absorption of H_2_O molecules and O_2_, which degraded the 2D MoS_2_/Ag NW electrodes. Therefore, by sputtering the PTFE film on the 2D MoS_2_/Ag NW, we demonstrated high quality PTFE/MoS_2_/Ag NW electrode for the high performance and operating stability of the TFHs because of the efficient passivation property and outstanding thermal dispersion ability. Even after the 85 °C–85% of temperature-relative humidity environment test, the 2D MoS_2_/Ag NW-based TFHs showed stable temperature profiles and repeated on/off properties, due to the effective passivation of the PTFE layer against the bare Ag NW-based, and 2D MoS_2_/Ag NW-based TFHs. Consequently, this certainly proposes that high quality PTFE film prepared by sputtering process provides effective thin film passivation for the 2D MoS_2_ and Ag NW hybrid electrode against external environment condition for advanced smart windows.

## Methods

### Fabrication of the MoS_2_-coated Ag NW electrodes

The MoS_2_ nanosheets were produced by electrochemical exfoliation. The MoS_2_ crystals (purchased from HQ graphene) were fixed with an alligator clip as a cathode, and placed with a graphite rod as a counter electrode. Tetra-heptyl ammonium bromide as an intercalant was dissolved in acetonitrile at a concentration of 5 mg/mL. The electrochemical reaction was attained with an applied voltage of 7 V for 1 h. After the reaction, the MoS_2_ crystals were cleaned with ethanol, and sonicated in 0.2 M polyvinylpyrrolidone in a dimethylformamide (DMF) solution for 30 min. To remove unexfoliated crystals, the as-prepared dispersion was centrifuged at 4000 rpm for 10 min. DMF was exchanged with isopropanol for spin coating. This MoS_2_ solution was spin-coated at 2500 rpm for 40 s, and repeated for 2 times on the Ag NW film that was fabricated by a roll-to-roll (RTR) slot-die coating system on polyethylene terephthalate (PET) substrate (TORAY ADVANCED MATERIALS KOREA INC.).

### RF sputtering of PTFE passivation layer

PTFE film was deposited using a 4-in. PTFE target (PTFE: 95 wt%, carbon nanotubes: 5 wt%) through an RF magnetron sputtering system. The PTFE films were deposited at the condition of constant RF power of 150 W, working pressure of 4 mTorr, and argon gas flow of 20 sccm. To improve the uniformity of the PTFE film, the substrate was rotated at a constant speed of 15 rpm, and maintained with cathode gun tilted at 30°. The PTFE film was deposited on the MoS_2_/Ag NW sample as a function of PTFE thickness from (50 to 200) nm, respectively.

### Fabrication of TFHs and test

Further, electrodes were deposited using a 4 in. AgPdCu target (APC; Ag: 99.90 wt%, Pd: 0.05 wt%, Cu: 0.05 wt%; Dasom RMS) through an DC magnetron sputtering system under 8.0 × 10^−7^ Torr base pressure. The APC electrodes were coated at the condition of constant DC power of 100 W, working pressure of 1 mTorr, and argon gas flow of 20 sccm. Also, the deposition time was the same at 250 s for all samples. We prepared a 2.5 cm × 2.5 cm Ag NW film sample, with APC electrodes of 0.6 cm × 0.6 cm at both ends of conductive sample. For APC deposition, a PET masking pattern having a size of 2.5 cm × 1.3 cm was attached to each sample consisting of the bare Ag NW, MoS_2_/Ag NW, and PTFE/MoS_2_/Ag NW. In consideration of the temperature range of the measuring equipment and the physical characteristics of the PET substrate, the upper limit of the measurement temperature was set at 120 °C. The applied voltage remained for 400 s until the saturation temperature. The saturation temperature was returned to the initial temperature through voltage turn-off for 200 s, and the test was repeated by slightly increasing the input voltage.

### Characterization of the PTFE/MoS_2_/Ag NW samples

The optical and electrical properties of the bare Ag NW, MoS_2_/Ag NW, and PTFE/MoS_2_/Ag NW were investigated by UV/visible spectrometer (UV540, Unicam), four-point probe (FPP-HS8, DASOL ENG), and Hall measurement (HMS-4000AM, Ecopia). To analyse the mechanical properties of different samples, we used the bending test system (JIRBT-620, JUNIL TECH) to investigate the critical radius and fatigue test. Field-emission scanning electron microscopy (FE-SEM: JSM-7600F, JEOL) was used to examine the surface morphology of the sample. High-resolution transmission electron microscopy (HR-TEM: JEM-2100F, JEOL) was used to analyse the interface between Ag NW and MoS_2_ or PTFE with the cross-sectional image. To calculate the surface energy of the bare Ag NW, MoS_2_/Ag NW, and PTFE/MoS_2_/Ag NW, we used the contact angle measurements (Phoenix-MT(A), SEO CO), using the liquid of deionized water and diiodomethane droplet. To evaluate the performance of the TFHs, temperature measurement system (McScience) examined the temperature of the sample using a contact thermocouple and an IR thermal imager (A35sc, FLIR, Wilsonville) with a thermal camera (FLIR ONE Pro) on the prepared sample on several DC voltage by Keithley 2634B. For the stability test of the fabricated TFHs, an environmental test under 85 °C–85% temperature-relative humidity was conducted for 140 h using the low temperature and humidity chamber (TH3-KE (Tabletop), JEIO TECH).

## Supplementary Information

Supplementary Figures.
