# Supplementary material for: Transparent and flexible passivation of MoS2/Ag nanowire with sputtered polytetrafluoroethylene film for high performance flexible heaters
Source: Sci Rep. 2022 Apr 9;12:6010. doi: 10.1038/s41598-022-09813-6 (PMC8994750; doi:10.1038/s41598-022-09813-6)
Supplement: Supplementary file 1 — Supplementary Figures. [file 41598_2022_9813_MOESM1_ESM.docx]

Supplementary

Transparent and flexible passivation of MoS_2_/Ag nanowire with sputtered Polytetrafluoroethylene film for high performance flexible heaters

**Seung-Gyun Choi, Hae-Jun Seok, Jihyun Kim, Joohoon Kang, Han-Ki Kim***

School of Advanced Materials Science and Engineering, Sungkyunkwan University, Suwon, Gyeonggi-do 16419, Korea

[**^*^hankikim@skku.edu**](mailto:*hankikim@skku.edu)


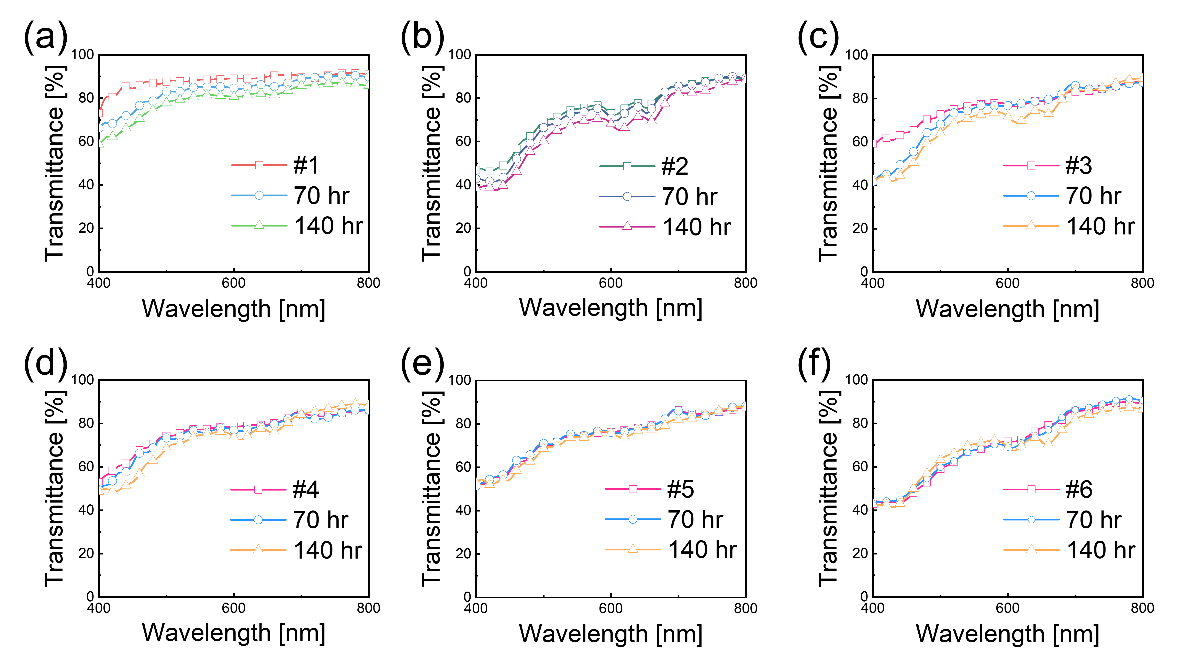


Figure S1. The optical transmittance of (a) the bare AgNW, (b) MoS_2_/Ag NW, and (c-f) PTFE/MoS_2_/Ag NW depending on time of the 85°C-85% environmental test.


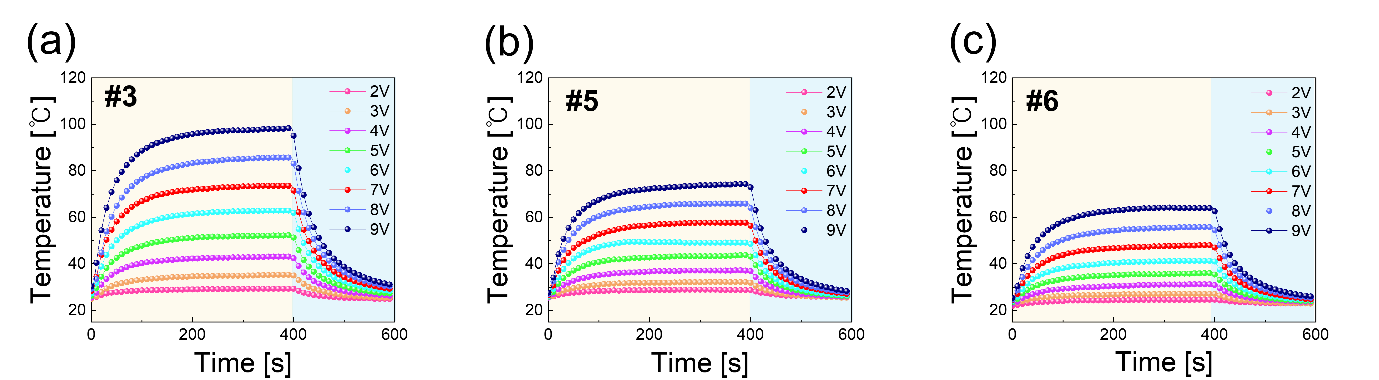


**Figure S2.** The performance of TFHs with various PTFE thickness of (a) #3: 50 nm, (b) #5: 150nm, and (c) #6: 200 nm as a function of DC voltage.


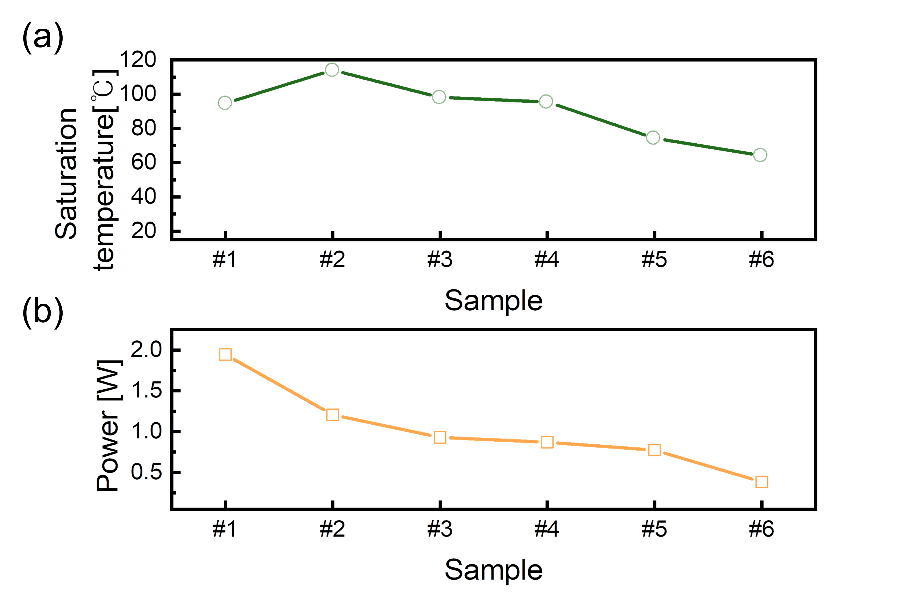


**Figure S3.** (a) The performance of TFHs for the saturation temperature, and (b) operation power value at DC 6V.


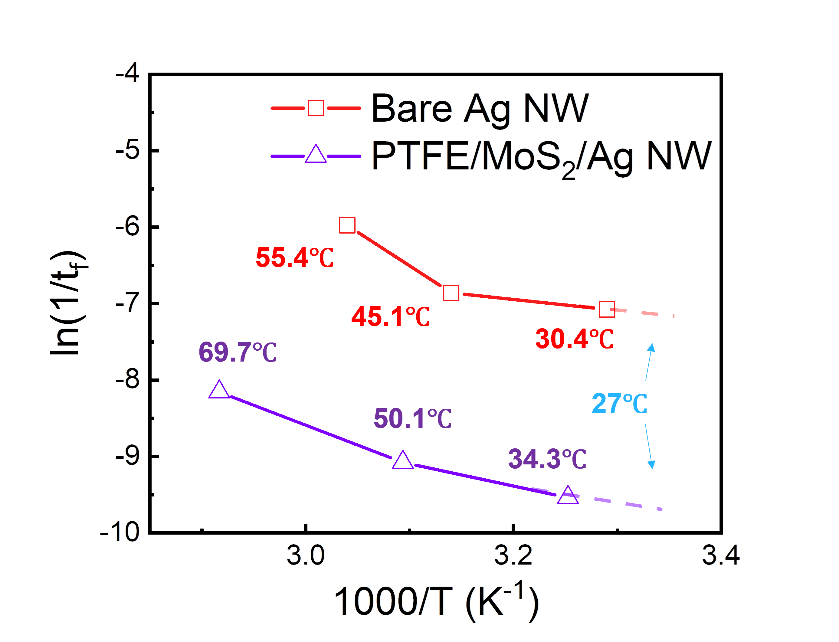


**Figure S4**. Arrhenius curve of the TFHs to evaluate the failure lifetime of devices under harsh environmental test
